# In-vitro evaluation of the effectiveness of polyphenols based strawberry extracts for dental bleaching

**DOI:** 10.1038/s41598-023-31125-6

**Published:** 2023-03-13

**Authors:** Shivani Kohli, Shekhar Bhatia, Spoorthi Ravi Banavar, Afaf Al-Haddad, Murugesh Kandasamy, Syed Saad Bin Qasim, Mak Kit-Kay, Mallikarjuna Rao Pichika, Umer Daood

**Affiliations:** 1 Restorative Division, School of Dentistry, International Medical University Kuala Lumpur, 126, Jalan Jalil Perkasa 19, 57000 Bukit Jalil, Wilayah Persekutuan Kuala Lumpur Malaysia; 2 Clinical Oral Health Sciences Division, School of Dentistry, International Medical University Kuala Lumpur, 126, Jalan Jalil Perkasa 19, 57000 Bukit Jalil, Kuala Lumpur Malaysia; 3 Department of Conservative Dentistry, Faculty of Dentistry, MAHSA University, Bandar Saujana Putra, Selangor Malaysia; 4 Division of Clinical Pharmacology, Department of Medicine, Indiana University, Indianapolis, 46202 USA; 5 Faculty of Dentistry, Department of Bioclinical Sciences, Kuwait University, 4th Ring Road, P.O Box 24923, 13110 Jabriya, Safat Kuwait; 6 School of Pharmacy, International Medical University Kuala Lumpur, 126, Jalan Jalil Perkasa 19, 57000 Bukit Jalil, Wilayah Persekutuan Kuala Lumpur Malaysia

**Keywords:** Microbiology, Chemistry

## Abstract

To formulate a dental bleaching agent with strawberry extract that has potent bleaching properties and antimicrobial efficacy. Enamel specimens (3 × 3 × 2 mm^3^) were prepared. Quaternary Ammonium Silane (CaC2 enriched) was homogenized with fresh strawberries: Group 1: supernatant strawberry (10 g) extract < Group 2: supernatant strawberry (10 g) extract + 15%HA (Hydroxyapatite) < Group 3: supernatant strawberry (10 g) extract + 15% (HA-2%k21) < Group 4: supernatant strawberry (20 g) extract only (20 g strawberries) < Group 5: supernatant strawberry (20 g) extract + 15% HA < Group 6: supernatant strawberry (20 g) extract + 15% (HA-2%K21) < Group 7: In-office Opalescence Boost 35%. Single-colony lactobacillus was examined using confocal microscopy identifying bacterial growth and inhibition in presence of bleaching agents using 300 µL aliquot of each bacterial culture. Images were analysed by illuminating with a 488 nm argon/helium laser beam. Colour difference (∆E00) was calculated using an Excel spreadsheet implementation of the CIEDE2000 colour difference formula and colour change measured between after staining and after bleaching. Scanning electron microscope was used to image specimens. Raman spectra were collected, and enamel slices were used for STEM/TEM analysis. HPLC was used for strawberry extract analysis. Nano-indentation was performed and X-ray photoelectron spectroscopy. Antioxidant activity was determined along with molecular simulation. hDPSCs were expanded for Alamar Blue Analysis and SEM. Mean colour change was significantly reduced in group 1 compared to other groups (*p* < 0.05). CLSM showed detrimental effects of different strawberry extracts on bioflms, especially with antimicrobial (p < 0.05). Groups 1, 2 and 3 showed flatter/irregular surfaces with condensation of anti-microbial in group 3. In strawberry specimens, bands predominate at 960 cm^−1^. HPLC determined the strawberry extracts content. Molecular simulation verified interaction between calcium and polyphenol components. XPS peak-fitted high-resolution corresponding results of Ca2p_3/2_ and Ca2p_1/2_ for all k21 groups. Combination of 10 g strawberry extract supernatant and 15% (hydroxyapatite 2%k21) improved the whiteness and provided additional antimicrobial potential. The novel strawberry extract and antimicrobial based dental formulation had immediate bleaching effect without promoting significant changes in enamel morphology.

## Introduction

Aesthetic demands are increasingly growing due to the society’s growing pressure for an appealing appearance. Dental bleaching is the most common aesthetic treatment option for lightening the colour of teeth beyond their natural shade in addition to other options of veneers and tooth-colored crowns^1–3^. The most common ingredient in these bleaching products is hydrogen peroxide along with its precursors, carbamide peroxide and other reactive oxygen species which causes the breakdown of large and dark chromophore molecules into smaller ones^4–6^. This process is based on a redox potential resulting in smaller molecules allowing greater reflection of light, leading to a whiter appearance of teeth along with improved chromatic and aesthetic features^7,8^. The safety of dental bleaching technique on the tooth structure has been questioned. Negative effects have been associated with dental bleaching and could be related to the pH value, oxidative effect, or composition of the bleaching agents^9^. Several unfavorable side effects can be reported, such as changes in the surface and translucency of enamel, dentin hypersensitivity, calcium depletion, demineralization, changes in organic structure, and surface hardness, pulp inflammation, and genotoxicity against odontoblast and collagen forming cells^10–15^. The toxicity increases with increasing concentration and duration of application of bleaching agents^16,17^.

Food habits such as consuming raw fruits and vegetables, have high amounts of organic acids which maintain and improve the tooth colour, making the use of this mechanism desirable. This enables developing a new natural tooth bleaching agent, with comparable aesthetic results and lower side effects^16^. The natural ingredients such as peroxide-free formulation (limonene and l-ascorbic acid present in citrus fruits, phthalimido peroxy caproic acid in vanilla fruits, lactic acid in dairy milk products, bromelain and papain in pineapple and papaya, actinidin in kiwi, banana peel and also strawberry) have been reported to cause teeth whitening effects. Since the natural ingredients are known to help whiten teeth, more understanding of these products is essential^18–21^.

Strawberry fruit is a rich resource for polyphenolic compounds (flavanols, i.e., glycosides of quercetin and kaempferol, esters of hydroxycinnamic acids, especially of p-coumaric acid, and ellagic acid and ellagic acid glycosides), tannins, proanthocyanins, and flavonoids which makes it an excellent antioxidant, anti-inflammatory, antimicrobial, and immunomodulatory agent^22^. It has been also reported to provide bleaching effect due to its acidic properties owing to presence of malic acid, citric acid and ellagic acid, working as a strong oxidizing agent on the enamel surface^17^.

Synthetic hydroxyapatite (HA) is a bioactive material that has been used to reduce the likelihood of demineralization in tooth bleaching pastes containing concentrated phosphoric acid and hydrogen peroxide^23^. However, the remineralization potential of HA may be reduced due to plaque accumulation and an acidic environment over the bleached tooth; thus, an antibacterial agent is required to enhance the properties of HA^24^. The organic compound quaternary ammonium silane (k21) has broad-spectrum antimicrobial activity with low cytotoxicity. In the presence of a long lipophilic alkyl chain, the antibacterial mechanism is based on bacterial cell wall lysis^25^. In the presence of a long lipophilic alkyl chain, the antibacterial mechanism is based on bacterial cell wall lysis^25^. Therefore, the aim of the study was to formulate an experimental dental bleaching agent composed of a strawberry extract containing HA merged with k21 antimicrobial. This was done to understand its composition, bleaching efficacy with surface effects, cytotoxicity, and antimicrobial potency. The null hypothesis was that there is no difference in cytotoxicity, antimicrobial, and bleaching efficacy between the commercial bleaching product and six different experimental strawberry extract gels.

## Results

The mean and standard deviation of ΔE00 and ΔWI_D_ are shown in Table 1. All bleaching solutions resulted in perceptible colour difference (ΔE00 > 0.8) that was clinically acceptable (ΔE00 > 1.8). Group 6 resulted in the highest mean of colour difference (ΔE00). The mean of colour difference was significantly less in group 1 compared to groups 2, 3, 6 and 7 (*p* = 0.04, 0.003, 0.00 and 0.04 respectively). There were no significant differences among other groups (*p* > 0.05). For ΔWI_D_, all bleaching solutions had a whiteness colour change and group 3 yielded the highest whiteness; however, no significant difference was observed among all the tests groups and control group.

**Table 1:** Data values for colour changes (ΔE00) and Whiteness (Δ WI_D_) after bleaching procedures. Lowercase for columns indicate significant differences with Bonferroni test (p < 0.05).

Group components	ΔE00 Mean (DS)	ΔWI_D_ Mean (DS)
10 g of strawberries dissolved in 50 mL of distilled water (supernatant) (Group 1)	4.32 (1.72)^ac^	8.76 (5.96)
10 g of strawberries dissolved in 50 mL of distilled water + 15% HA (supernatant) (Group 2)	8.79 (2.47)^bc^	12.19 (8.22)
10 g of strawberries dissolved in 50 mL of distilled water + 15% (HA-2%K21) (Group 3)	9.93 (3.69)^bc^	18.86 (8.78)
20 g of strawberries dissolved in 50 mL of distilled water (supernatant) (Group 4)	7.31 (3.86)^c^	15.02 (10.88)
20 g of strawberries dissolved in 50 mL of distilled water + 15% HA (supernatant) (Group 5)	7.79(3.21)^c^	10.45 (11.33)
20 g of strawberries dissolved in 50 mL of distilled water + 15% (HA-2%K21) (supernatant) (Group 6)	11.80 (3.14)^bc^	7.55 (13.87)
In-office bleaching product—Opalescence Boost 35% (Group 7)	8.79 (3.23)^bc^	14.59 (14.22)

**Figure 1:**
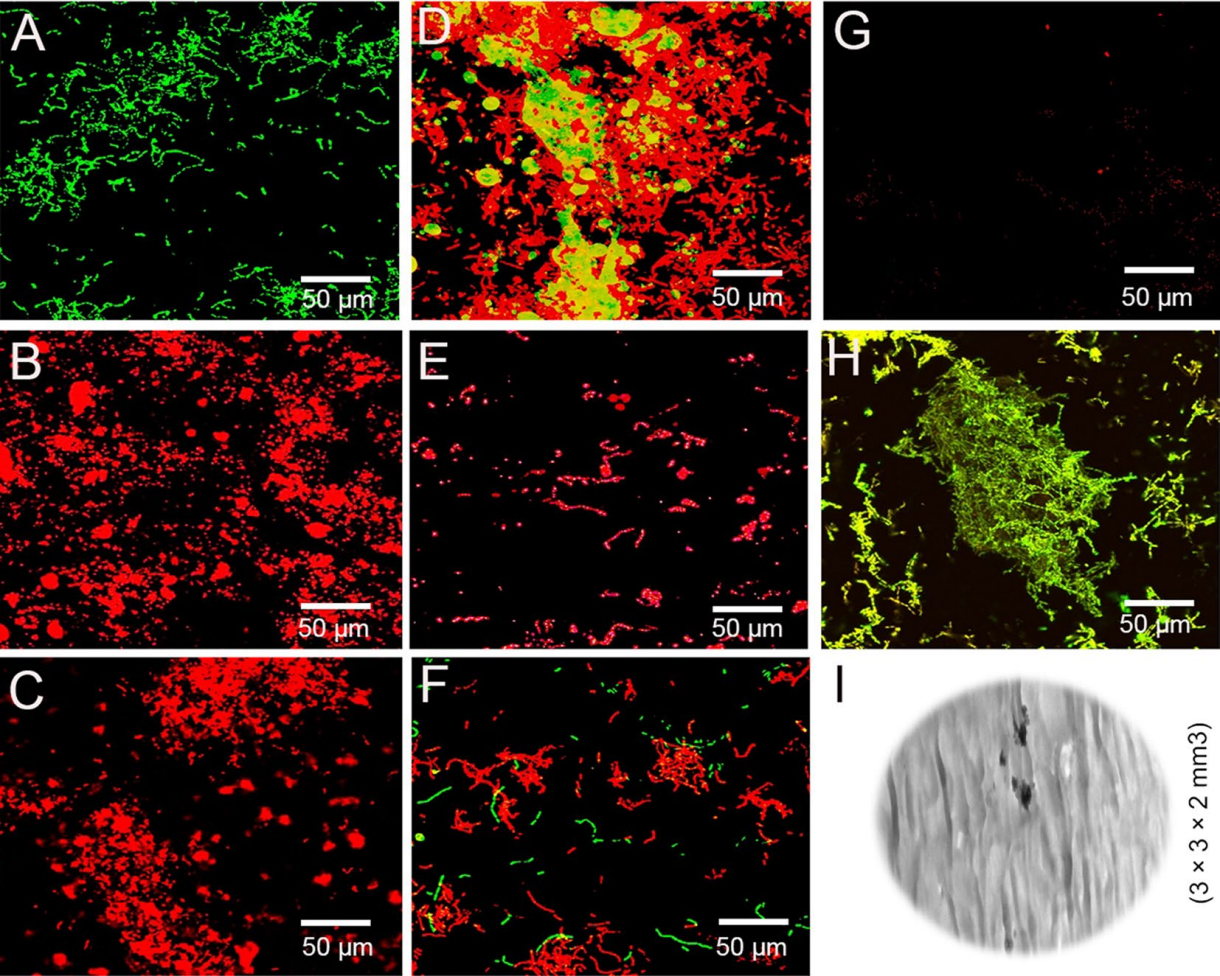
CLSM images and viability of single specie lactobacillus biofilms treated with different antimicrobial agents, after which, biofilms were stained using the BacLight LIVE/DEAD viability stain. CLSM images showing (**A**) control bacterial sites without any application of bleaching aids. (**B**) Group 1; (**C**) Group 2; (**D**) the k21 molecule QAS is attracted due to its surfactant effect towards bacterial colonies in Group 3; (**E**) Group 5; (**F**) Group 4; (**G**) Group 6; (**H**) Group 7; (**I**) enamel surface used for biofilm growth.

CLSM images are representative of single species lactobacillus seen in Fig. 1 depicts effects of different strawberry extracts on bioflms. The control specimens in BHI medium (Fig. 1-A) showed clusters of green colonies as the majority of the dead bacterial cells formed aggregates and were found in all the strawberry experimental group specimens. The concentration of strawberry extract used affected aggregation (p < 0.05) and percentage of dead bacteria. In contrast to the control, the 10/20 g specimens exhibited different characteristics, forming thinner biofilms with no distinct aggregates, as the concentration of strawberry was increased, indicating a dominant role for bacterial biofilm removal and non-adherence while performing the bleaching procedure (Table 2). Because of the surfactant effect of k21 molecules (this is discussion) within the group 3 specimens, the dead bacteria formed dead isolates around the k21 molecule. The live cell number was significantly higher in the control specimens compared to the specimens of group 3 and 6 with k21, which is consistent with the inability to form biofilm (Table 2), with the least percentage formed within the opalescence specimens.

**Table 2:** Bacterial viability in single-species lactobacillus biofilms following different bleaching treatments. Values are means ± standard deviation in percentages. bioimageL software (version 2.0, Malmö, Sweden; www.bioimage.org) was used to examine the fluorescent images of the biofilm at different stacks. Groups identified by different letters (in columns) were significantly different at p < 0.05.

Specimen	Mean percentage % dead bacteria	Mean percentage % live bacteria
10 g of strawberries dissolved in 50 mL of distilled water (supernatant) (Group 1)	81.2% (± 9.8) a	18.8% (± 4.1) a
10 g of strawberries dissolved in 50 mL of distilled water + 15% HA (supernatant) (Group 2)	79.5% (± 7.9) a	20.5% (± 3.3) a
10 g of strawberries dissolved in 50 mL of distilled water + 15% (HA-2%K21) (Group 3)	91.4% (± 12.3) b	8.6% (± 2.5) b
20 g of strawberries dissolved in 50 mL of distilled water (supernatant) (Group 4)	72.3% (± 5.6) a	27.7% (± 5.3) a
20 g of strawberries dissolved in 50 mL of distilled water + 15% HA (supernatant) (Group 5)	75.8% (± 10.1) a	24.2% (± 6.3) a
20 g of strawberries dissolved in 50 mL of distilled water + 15% (HA-2%K21) (supernatant) (Group 6)	96.5% (± 8.5) b	3.5% (± 0.9) b
In-office bleaching product—Opalescence Boost 35% (Group 7)	62.7% (± 13.2) c	37.3% (± 6.6) c

**Figure 2:**
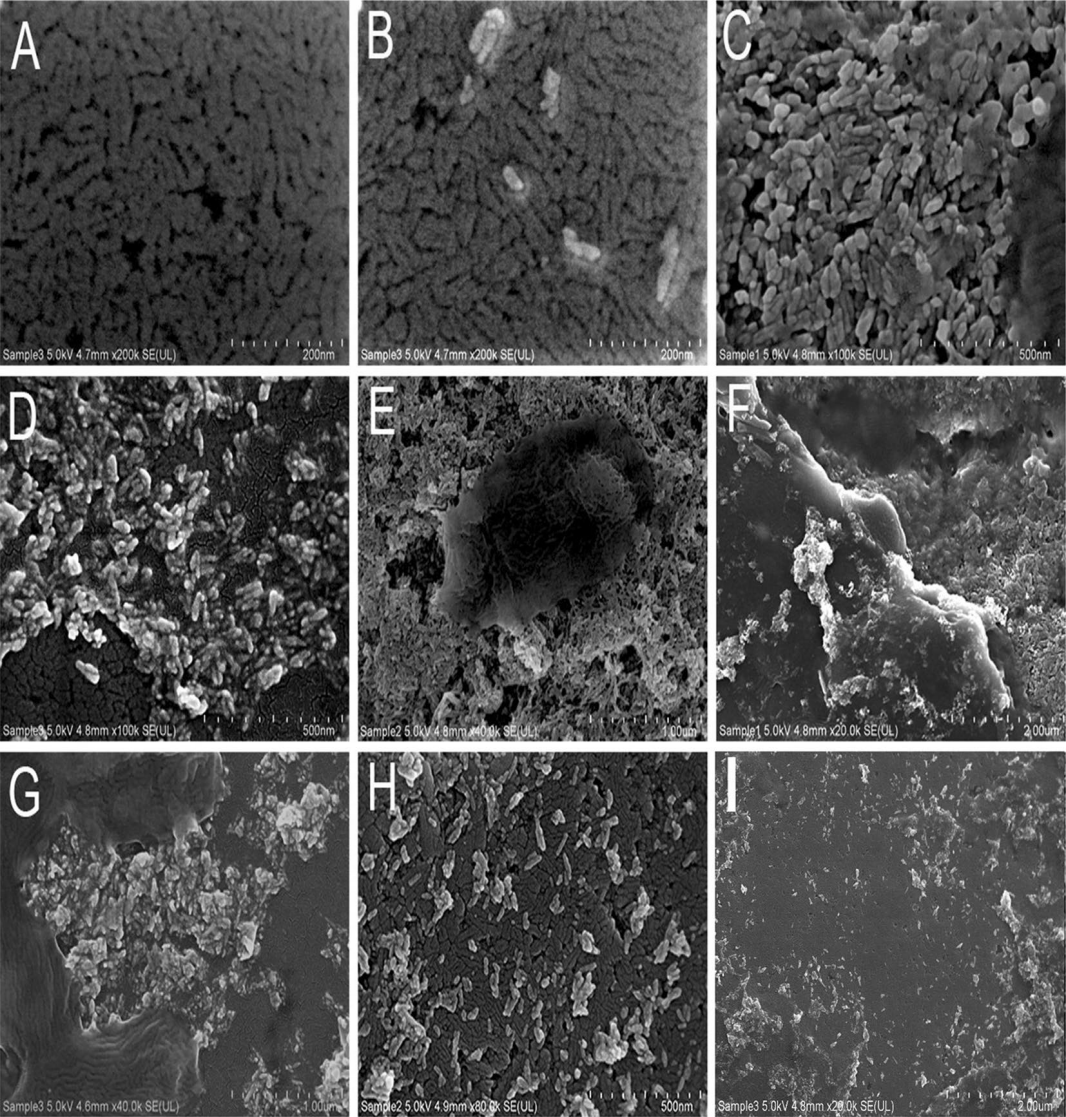
Representative FE-SEM micrographs of enamel specimens observed under the experimental conditions of (**A**, **B**) normal enamel surface; (**C**) Group 1; (**D**) Group 2; (**E**) Group 3; (**F**) Group 4 showing a thickened layer; (**G**) Group 5; (**H**) Group 6 (**I**) Group 7.

The images obtained by scanning electron microscopy analysis (Fig. 2) revealed irregularities on the enamel surface after different bleaching treatments. The 10 g strawberry (Fig. 2-C) showed changes on the enamel surface compared to those of the untreated group (Fig. 2A,B). The images of group 1, 2 and 3 (Fig. 2C–E) showed flatter and irregular surfaces with condensation of k21 molecules on top of the enamel surface seen in group 3. Similar intermediate changes in the surface were also seen in the 20 g strawberry group compared to the untreated enamel with folds of strawberry extract coating evident in group 4 (Fig. 2F) and group 5 (Fig. 2G). The images obtained by scanning electron microscopy analysis (Fig. 2I) revealed massive irregularities in the enamel surface when the specimens were submitted to treatment with Opalescence Boost 35% (group 7).

**Figure 3:**
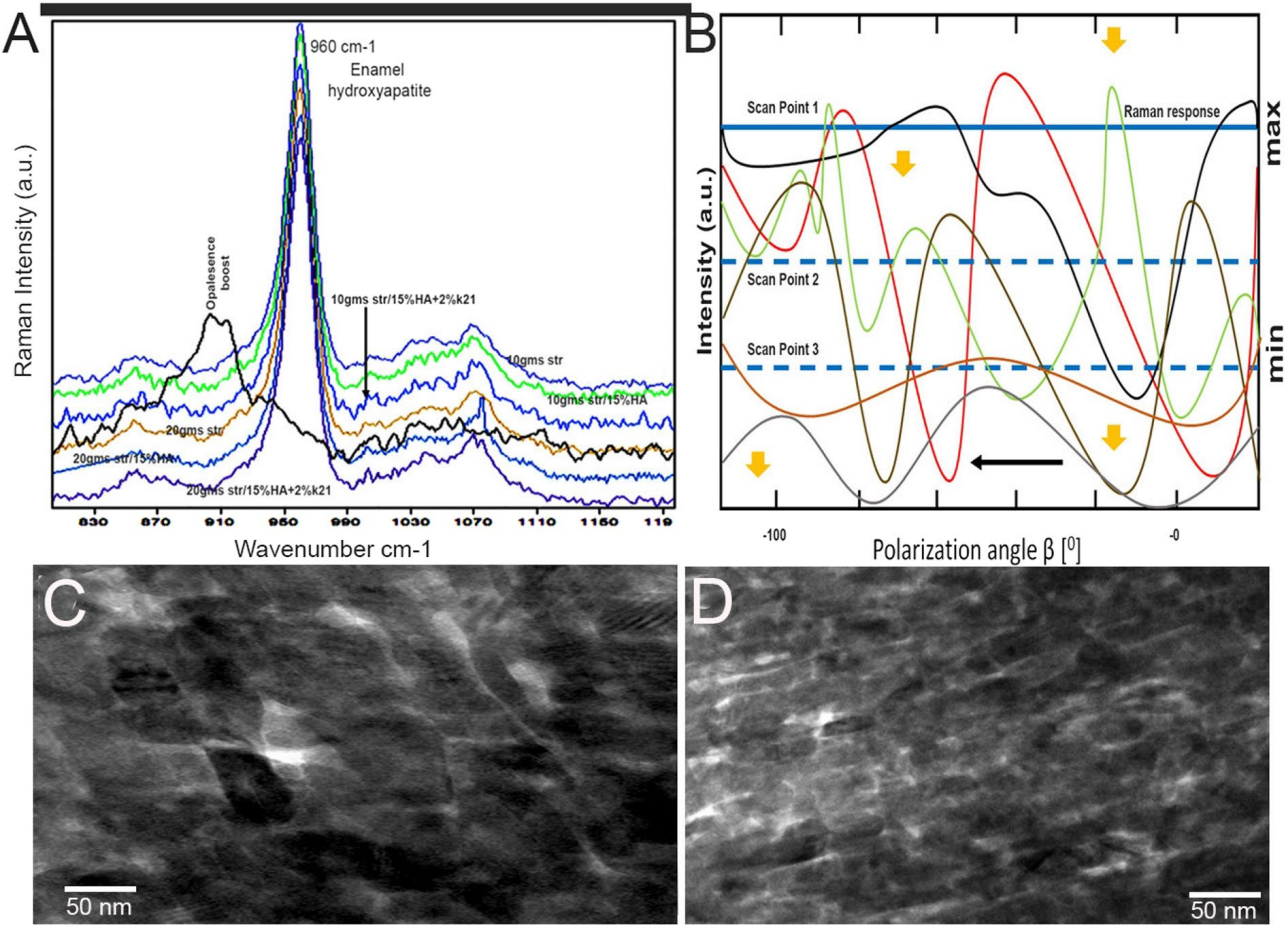
(**A**) Raman spectra of treated and control enamel specimens with different concentrations of strawberry extracts (control data not shown). Spectral differences of control and treated specimens can be seen in the 960 cm^−1^ regions after normalization. Labelled bands present in the spectra are discussed in the text. Spectra are shifted to avoid overlap between the groups. The spectral lines are quantitative detection with each data point corresponding to the average signal collected from different groups. For better comparability of the two measurements, different colours were chosen for the Raman spectrum. (**B**) The hydroxyapatite orientation map visualization of different enamel specimens (**C**, **D**) crystal orientation of specimens treated with Group 3 and Group 6 respectively.

**Figure 4:**
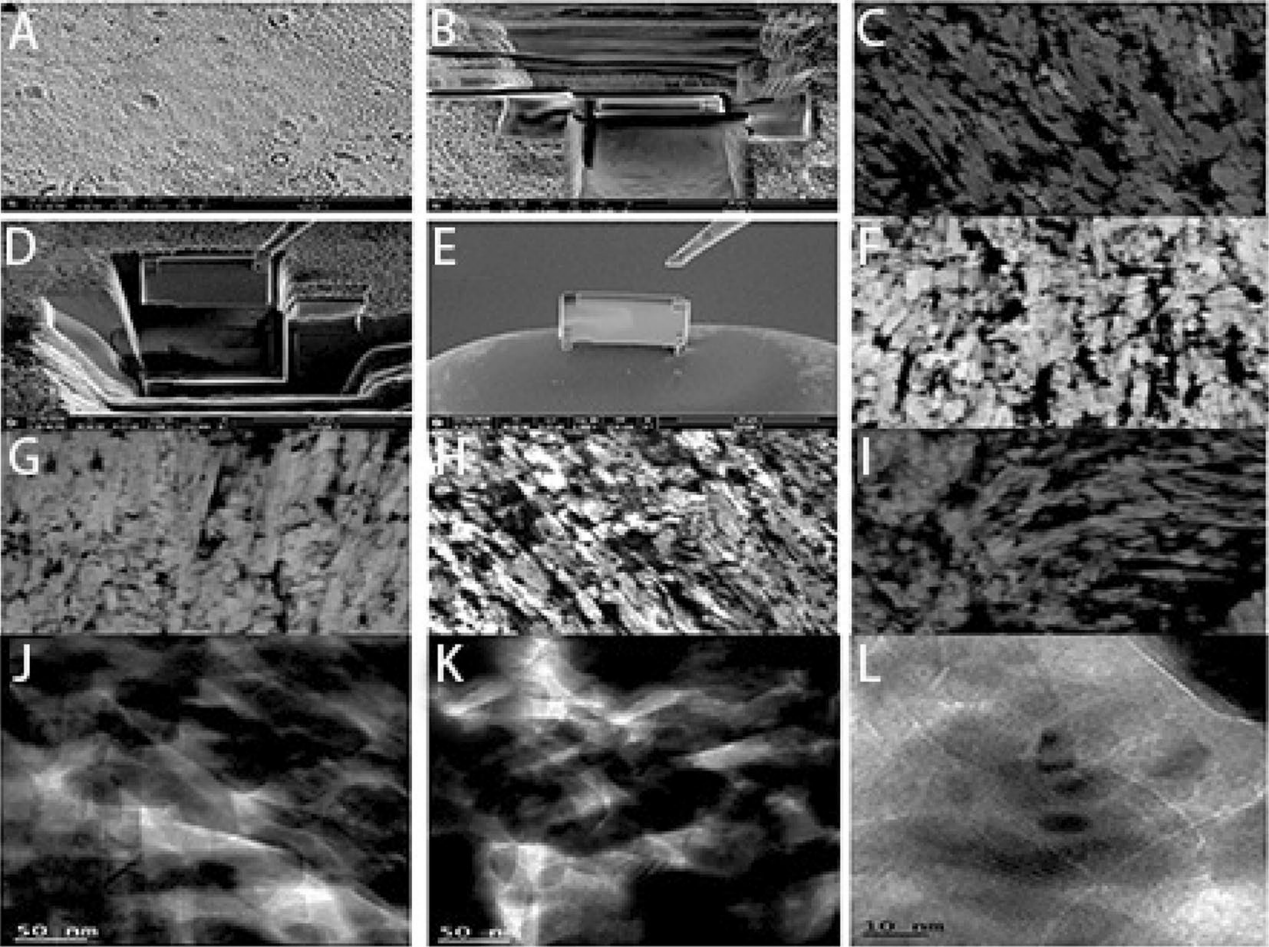
(**A**–**D**) Representative micrograph of FIB-milled cantilever with a notch and bending overview (the sample is tilted 25^0^ with respect to the electron beam within the prisms avoiding the prism sheaths. The samples consisted of HAP(HA) crystallites only, representing the hierarchical level 1. Loading was achieved by using the optical microscope of the nanoindentation system, and the Berkovich tip was positioned at the cantilever’s free end, as marked by the arrow. (**E**–**I**) STEM image with high magnification resolution resulting in approximately 0.6 px per nm showing prism boundaries with mineral-on-mineral contact. The shapes of the crystallites often fit together like puzzle-pieces and seem to be interlocking (circled areas) but in some cases there are larger gaps present between the crystallites depending on the treatment; (**E**) no application specimens; (**F**) Group 2; (**G**) Group 3 (**H**) Group 5 (**I**) Group 6 (**J**) TEM image showing the HAP crystallites in perpendicular orientation to the image plane in Group 3. The lattice planes of the crystallites are visible. Crystallite boundaries manifest either as a thin white line or can be distinguished by the different orientations of the lattices the crystallites. (**K**) It can be seen that crystallites are overlapping each other and, in some cases, seem to be in contact with each other in Group 6. (**L**) The darker spots appearing all over the picture are broken crystal lattices due to the damage caused by In-office bleaching product—Opalescence Boost 35% in Group 7.

In strawberry exposed specimens, bands associated with the inorganic phase predominate at the 960 cm^−1^ fingerprint region (Fig. 3A). The PO or phosphate stretching within the enamel mineral is identified. The most intense mineral characteristic bands are hydroxyapatite symmetric phosphate stretching at different intensity levels for different specimens. In Fig. 3, the maximum spectral bands for the measured 960 cm^−1^ spectrums are shown with minor shifts. The intensity of the 10 g strawberry levels in distilled water specimens increased (Fig. 4). The intensities decreased with an increase in strawberry concentration and k21/HA additions as seen in group 5 and 6. The change in intensity ratio approximating for Opalescence specimens clearly demonstrated differences as compared to other experimental specimens (p < 0.05), showing a remarkable decrease in intensity (Table 1). In Fig. 3B, the oscillatory mean 960 cm^−1^ intensity reflects the characteristic enamel structure for control and experimental specimens. All specimens show measurement yielding a precise sinusoidal response of polarization angle. Based on the results, the Raman mapping corresponded to a full rotation φ = 0° to φ = 180°. The entire mapping was created by fitting a 960 cm^−1^ Raman band collected at various polarization angles. Relative intensities of hydroxyapatite increased with decreasing strawberry concentration showing sharp changes (Fig. 3B). The crystal orientation of specimens in group 3 and group 6 can be seen following the curvature of the prism boundary where the prisms seem to be neatly stacked on top of each other (Fig. 3C,D). All the results, including the peak area percentages are shown in Table 3, which are basically consistent with the other Raman results. However, for commercial Boost group, peak area results obtained on basis of Raman spectra were lower in presence of some experimental error.

**Table 3:** Antioxidant obtained from DPPH, Ascorbic acid and Trolox as standards with total antioxidant capacities of different groups. Means ± standard deviations of the variations in the apparent bulk elastic modulus (Eappr) after bleaching treatments. Raman spectra with relative peak areas for 960 cm^−1^ and XPS data recorded in the enamel crystal shift range for different groups. XPS data was analysed using CasaXPS Software (Casa Software Ltd, United Kingdom); version 2.3.25; http://www.casaxps.com/. Different upper/lowercase letters with symbols are statistically significant at p ≤ 0.05.

Groups for antioxidant analysis						
DPPH (**34.1 ± 3.9)**	Ascorbic acid **(30.1 ± 7.3)**	Trolox **(39.11 ± 5.1)**	Apparent Elastic Modulus (E_appr GPa_)	Raman enamel crystal shift range (cm^−1^)	XPS Ca2p_3/2_ Ca2p_1/2_ eV	Relative Peak area (%)
10 g of strawberries dissolved in 50 ml of distilled water (supernatant) (Group 1)	31.32 ± 6.7 a		110.85 ± 6.8 A	960 cm^−1^ γ	321.1 θ	0.051 A
10 g of strawberries dissolved in 50 ml of distilled water + 15% HA (supernatant) (Group 2)	32.1 ± 3.3 a		107.11 ± 6.1 A	961 cm^−1^ γ	356.7 φ	0.043 A
10 g of strawberries dissolved in 50 ml of distilled water + 15% (HA-2%K21) (Group 3)	33.2 ± 8.7 a		104 ± 9.8 A	960 cm^−1^ γ	341.3 θ	0.066 A
20 g of strawberries dissolved in 50 ml of distilled water (supernatant) (Group 4)	45.4 ± 8.1 b		98.85 ± 11.7 AB	960 cm^−1^ γ	333.6 θ	0.063 A
20 g of strawberries dissolved in 50 ml of distilled water + 15% HA (supernatant) (Group 5)	47.1 ± 11.1 b		101 ± 12.1 A	961 cm^−1^ γ	349.3 φ	0.071 A
20 g of strawberries dissolved in 50 ml of distilled water + 15% (HA-2%K21) (supernatant) (Group 6)	42.1 ± 9.9 b		96 ± 10.1 AB	959 cm^−1^ γ	343.1 θ	0.09 B
In-office bleaching product—Opalescence Boost 35% (Group 7)	36.4 ± 4.9 c		71.9 ± 12.1 C	956 cm^−1^ δ	299.8 Δ	0.04 C

The arrangement of prismatic and interprismatic enamel varies with bleaching agent treatment at lower and higher magnifications. In both group 3 10 g (Fig. 3C) and group 6 10 g (Fig. 3D), the prisms appear to be neatly stacked on top of each other in the innermost and inner enamel (Fig. 3D). The prism boundaries as observed within the outer enamel appear open having connections with the interprismatic enamel. There was a discontinuity observed within the prism boundaries for 20 g specimens (Fig. 3D). As observed in Fig. 4, the STEM image identifies the cross-section of the crystallites found in a layer of interprismatic and prismatic enamel after modification with carious bleaching agents (Fig. 4A–I). The observed crystallites are mostly ambiguous polygonal shapes in cross-section. STEM image with a high magnification resolution of about 0.6 px per nm displaying prism boundaries with mineral-on-mineral contact. The crystallite shapes often fit together like puzzle pieces and appear to be interlocking, but depending on the treatment, there are larger gaps present between the crystallites. Figure 4E is an example of a no-application specimen with smoother enamel than the other groups (Fig. 4F–I).

The TEM images for group 3 identified HAP crystallites perpendicular to the image plane with visible lattice planes. The boundaries were characteristically thin white observable lines with crystal lattices having different orientations. Group 6 (Fig. 4K) was determined with overlapped crystallites and in contact with one another in some of the observed areas (Fig. 4L). The darker spots were seen to appear as broken crystal lattices due to the damage caused by In-office bleaching product—Opalescence Boost 35% in group 7.

**Figure 5:**
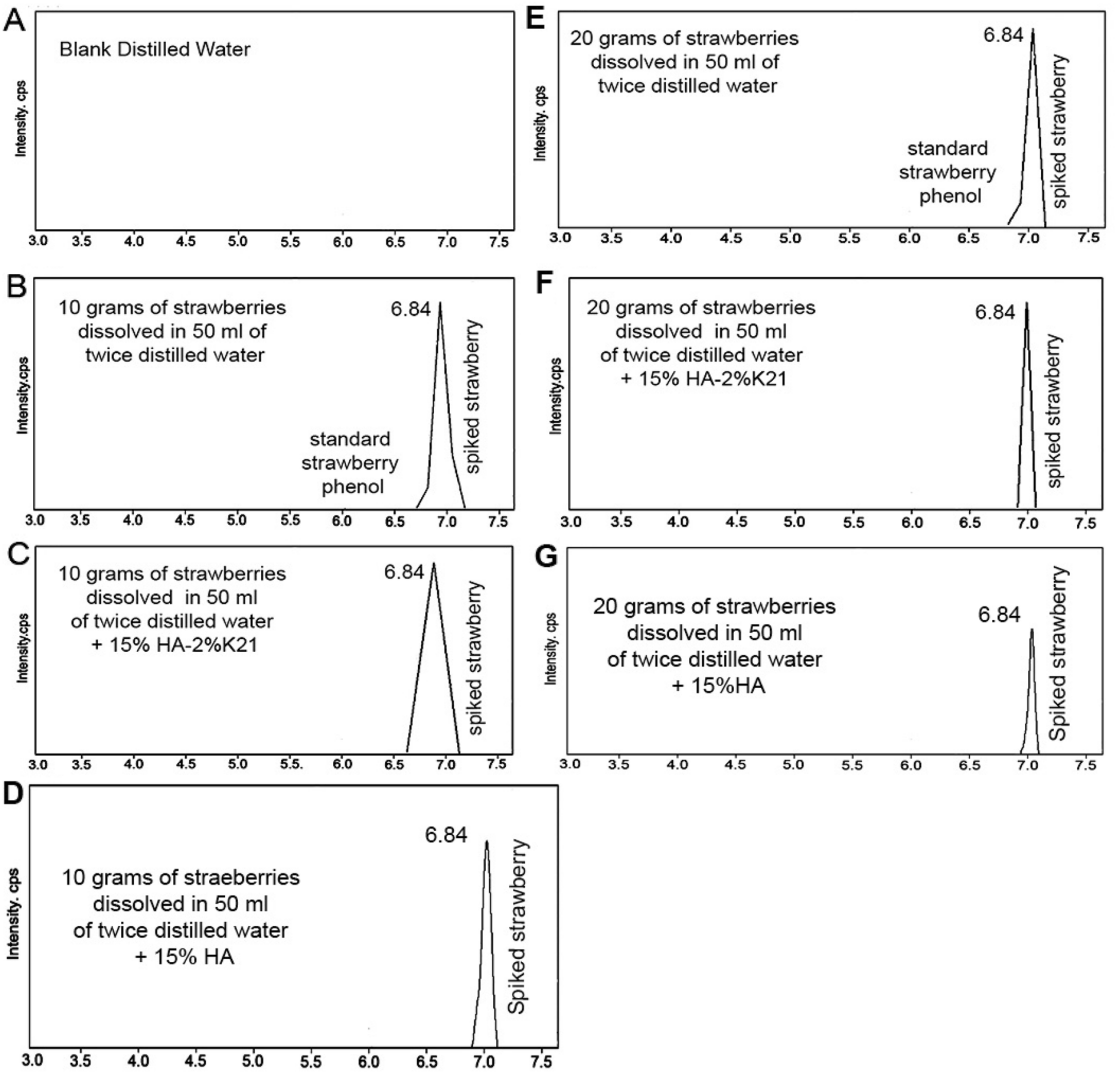
HPLC of experimental extracts; (**A**) Blank solvent; (**B**) Group 1; (**C**) Group 3; (**D**) Group 2; (**E**) Group 4; (**F**) Group 6; (**G**) Group 5.

The retention time threshold for liquid chromatography (Fig. 5) was met in this study. Overall, the HPLC analysis method employed the final determination. The method met the requirements for specificity, repeatability, and limit of quantitation, with higher intensities seen in 20 g strawberry extracts. All strawberry spikes were identified in all phenol groups (Fig. 5).

**Figure 6:**
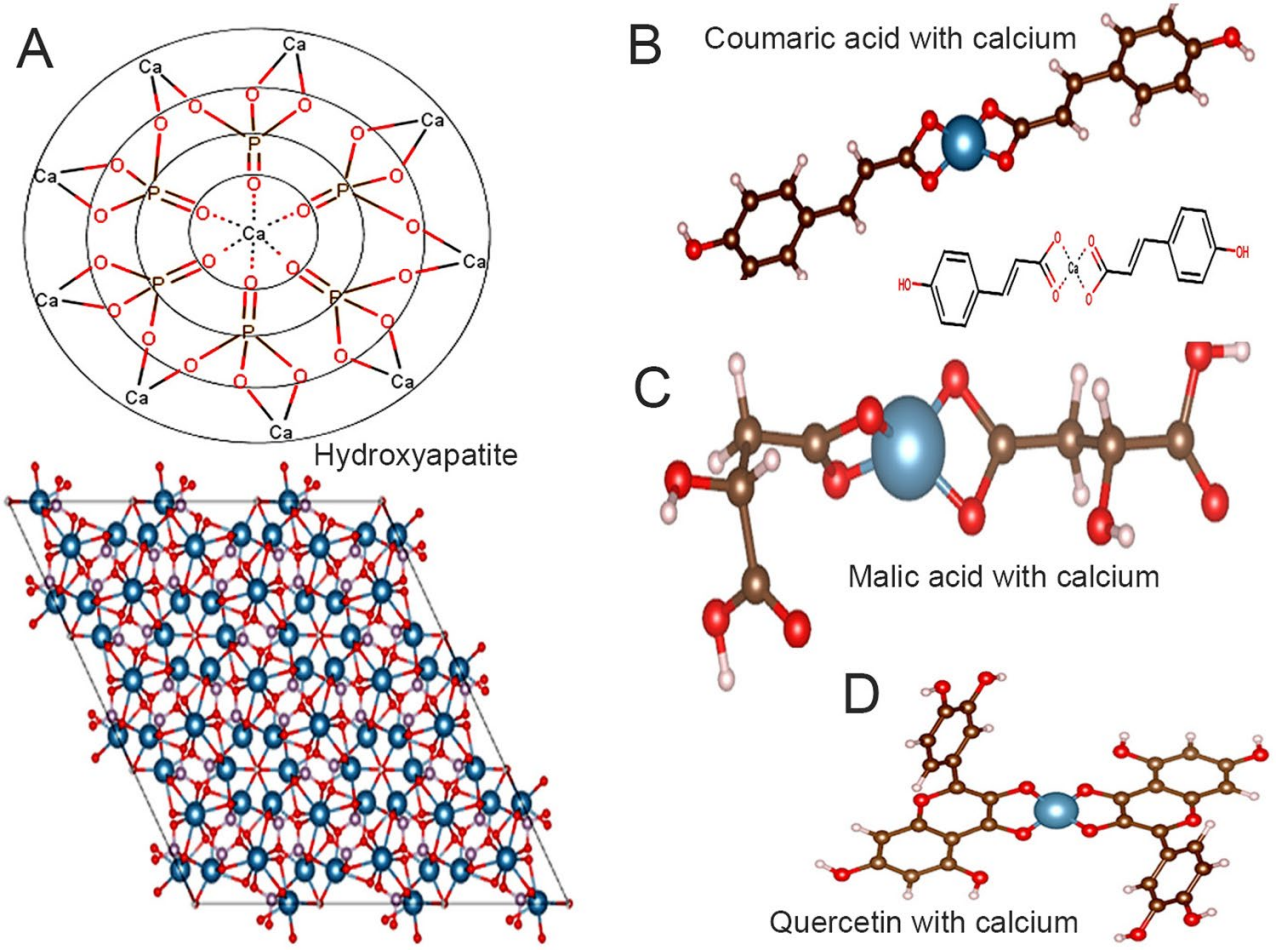
Alignment of Asymmetric unit of hydroxyapatite and polyphenol set molecules acquired from Marvin, ChemAxon database using VESTA 3 with molecular structure and predicted activity of the three eligible compounds with the enamel structure (with the atomic labeling scheme.). (**A**) 2D and 3D diagrams of the hydroxyapatite molecules; (**B**–**D**) The position and the 3D interactions of coumaric, malic and quercetin acids with calcium of the hydroxyapatite into the binding pocket with docked pose. The diagram is depictive of layers approximately parallel to the (− 120) plane.

The molecular simulation analysis also verified the interaction between the calcium of the hydroxyapatite and polyphenol strawberry components (Fig. 6). The hydroxyapatite molecule (Fig. 6A) structure conformations analysis obtained from Marvin, ChemAxon showed extended strands and geometric arrangement with radial distribution of calcium molecules. The hydroxyapatite calcium (Fig. 6B) was close to the (001) of polyphenol compound crystal planes, indicating that they were attracted to each other, which could describe the hydroxyapatite-polyphenol interfacial structure. An electrovalence bond was formed between the positively charged Ca^2+^ on the HAP crystal face and the negatively charged O atom of the polyphenol acid group. The Ca–O coordination interaction was the most important in determining HAP's binding affinity to the phenol acids, but there was also some van der Waals force interaction (Fig. 6C,D).

Table 4 depicts the corresponding peak positions of the high resolution XPS performed for quantifying Ca2p_3/2_ and Ca2p_1/2_ for the experimental groups. The In-office bleaching agent—Opalescence Boost 35% represented the lowest corresponding peaks (299.8 eV) for the P–O bonding of the enamel surface. The higher peak corresponding to the Ca2p_3/2_ and Ca2p_1/2_ peaks were found for all strawberry groups except group 1. The relative intensity ratio was corrected as the peaks are separated by 1.8 eV. The experimental strawberry groups Ca/P ratio (data not shown) atomic ratios were calculated and appeared above the expected values.

**Table 4:** Width measurements of hDPCs after the effect of bleaching application methods on size. n = number of measurements. The diameters of dental pulpal cells were measured using the Image J (ImageJ bundled with Java 1.8.0_172; database BisQue; https://imagej.nih.gov/ij/download.html) straight line tool package.

Specimen	Width (nm)	n	Relative Size
10 g of strawberries dissolved in 50 ml of distilled water (supernatant) (Group 1)	569.1 ± 55.9	3	87.1% α
10 g of strawberries dissolved in 50 ml of distilled water + 15% HA (supernatant) (Group 2)	512 ± 61.7	3	80.3% α
10 g of strawberries dissolved in 50 ml of distilled water + 15% (HA-2%K21) (Group 3)	575 ± 50.9	3	92.1% α
20 g of strawberries dissolved in 50 ml of distilled water (supernatant) (Group 4)	453 ± 33.5	3	84.0% α
20 g of strawberries dissolved in 50 ml of distilled water + 15% HA (supernatant) (Group 5)	423.6 ± 44.1	3	76.3% β
20 g of strawberries dissolved in 50 ml of distilled water + 15% (HA-2%K21) (supernatant) (Group 6)	444 ± 41.6	3	76.9% β
In-office bleaching product—Opalescence Boost 35% (Group 7)	399.6 ± 34.4	3	69.4% Δ

The antioxidant activity was performed to analyse the comparison with the standards of ascorbic acid and Trolox. The complementary assays of both strawberry extract and standards showed a dose-dependent increase in antioxidant activity with the least activity seen in group 1 specimens (Table 3).

**Figure 7:**
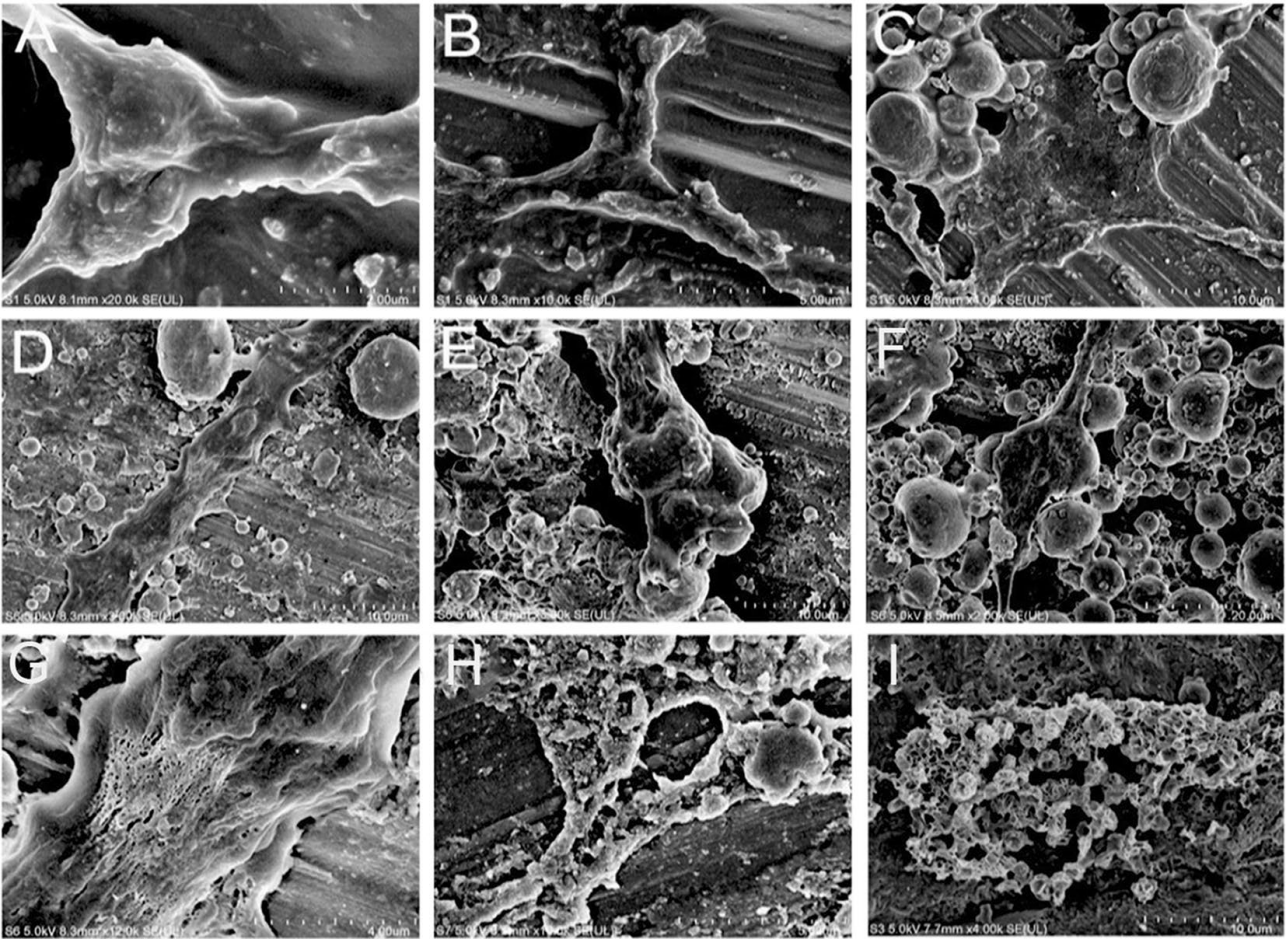
Scanning electron microscopy (SEM) of dental pulp stem cells (DPSCs) loaded onto enamel surface and treated with different bleaching agents. Different specimen groups are represented which show gold coated at high magnification; (**A**, **B**) control cells with no application of extracts; (**C**) Group 1; (**D**) Group 2; (**E**) Group 3; (**F**) Group 4 (**G**) Group 5; (**H**, **I**) Group 7.

The hDPCs seeded on the enamel specimens are presented in Fig. 7. The cells were seeded after exposure to different experimental bleaching agents. The cell morphology did not reflect any discernible changes between the control (Fig. 7A) and the strawberry groups (Fig. 7C–H). The cells resembled fibroblasts, as the cells grew on the enamel surface with dendritic cell extensions. Cells in the control and group 3 were flattened with large cytoplasm and attached to the enamel via multiple small cytoplasmic projections (Fig. 7) except for group 6 which showed cytoplasm thinning. In contrast, the opalescence treated cells (group 7) had irregular cells with few cytoplasmic processes and continuous blebs within the cell cytoplasm losing their fibroblast-like morphology. The number of cells that remained adhered to the enamel surface decreased progressively with increasing concentration of strawberry extract. The cell measurement method presented in this study is quick and reproducible. Artefacts such as shrinkage were seen amongst cells, especially in the opalescence groups showing shear thinning (Table 4). We propose that this observed difference is largely the result of loss of cell turgor.

**Figure 8:**
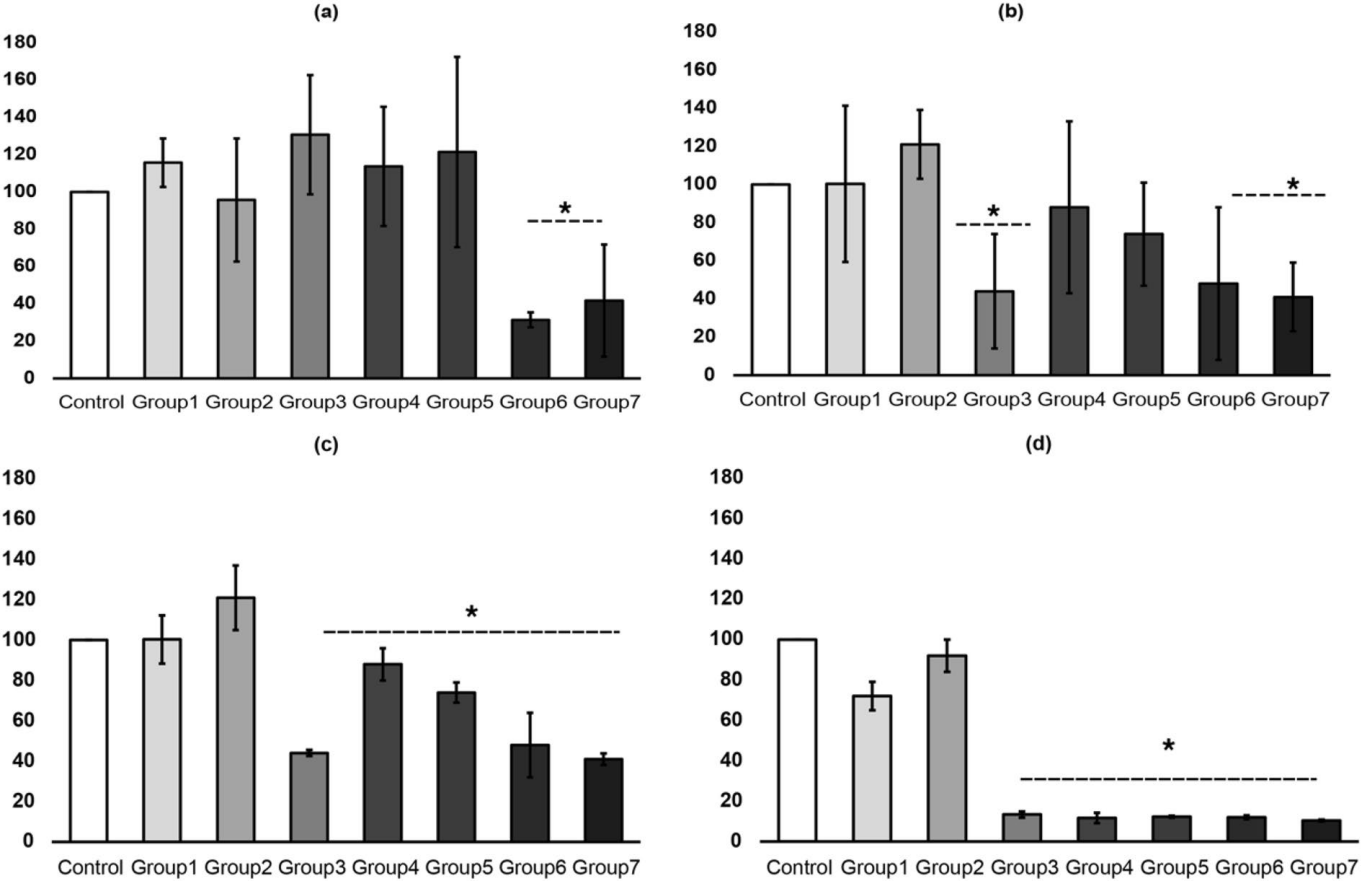
Graphs depicting the viability assay. The figures labelled (**A**–**D**) represent the mean ± standard deviations viable cells for each group. The cells were exposed for 1, 6, 12, and 24 h. The absorbance was read at 570–600 nm wavelength. *p values < 0.05 indicating significant differences between other groups at various time intervals.

The mean cell proliferation rates of the hDPSCs treated with different formulations (Fig. 8A–D) of strawberry extracts were compared to untreated cells using ANOVA and post-Hoc Tukey tests. Cell viability was decreased in groups 3, 4, 5, 6, and 7 in the dose and time-dependent manner. Groups 6 and 7 had significant effects on cell viability compared to other groups. Group 1 and 2 showed the best results with the least effects on cell viability at all four time points. ANOVA analysis showed significant differences between the groups at different time intervals of 1 h, 6 h, 12, and 24 h with p values of 0.010, 0.042, 0.00, and 0.00, respectively. Further, when Post-hoc Tukey was employed at the end of 1 h, group 6 and group 7 showed a significant reduction (52% and 59% respectively) in cell viability (p < 0.05) compared to control. Cells cultured in other formulations did not show significant changes compared to control. Further, when the cells were analysed after 6 h, cells cultured under formulations of groups 3, 6, and 7 showed decreased viability compared to untreated cells (p < 0.05). Cell viability in Groups 1, 2, 4, and 5 was not statistically different from control cells. At the end of 12 and 24 h, cells in groups 3, 4, 5, 6, and 7 showed a significant reduction in cell viability compared to control, group 1 and 2 (p < 0.05).

## Discussion

Despite the high efficacy of chemical bleaching gel, their biological or surface side effects are usually a concern^16^. In the current study, natural polyphenolic-based bleaching solutions (strawberry extracts) with remineralizing and antibacterial agents were assessed as potential materials for biocompatible and less destructive bleaching methods. Both colour change and whiteness index were used to assess their bleaching efficacy. ΔE00 use engaged a more accurate colour assessment as compared to the CIELAB system^26^. This is due to the fact that ΔE00 added up five corrections leading to a complete resolve of the uniformity issue including lightness (SL), chroma (SC), a hue rotation term (Rt), hue (SH) and compensation for neutral colours (L*C*h). The current study took each parameter into account while determining the efficacy of the experimental bleaching protocols investigated. In the study, only the **ΔWI**_**D**_ formula gives the performance value that is comparable to that of the average visual performance, and we therefore conclude that only this formula can be said to be acceptable in the context of practical use.

All bleaching solutions have perceptible colour change that indicated their bleaching efficacy. However, this efficacy was observed at different levels. The colour difference after bleaching caused by 10 g strawberry supernatant extract displayed colour changes were in agreement with the previous study^27^. However, the changes of colour (whiteness) resulted from 10 g strawberry along with HA and k21 was more favourable but not significant as a bleaching agent when compared to positive control group of Opalescence rejecting the null hypothesis. Adding hydroxyapatite and k21 produced significantly more colour change and more favourable whiteness. The attribution to a particular reason is speculative and needs further investigations. Furthermore, maintaining the concentration of strawberry to 10 g in group 3 resulted in high colour changes and favourable whiteness. This primarily maybe due to an increased release of hydroxyl free radicals which have significant bleaching effect^16,17,25^. On the contrary, adding hydroxyapatite and k21 to 20 g of strawberry extract had less favourable whiteness although more colour difference was produced. This phenomenon should help to understand that from the patients’ perspective, an effective whitening can be achieved with 10 g of strawberry content, which should be tested for good stability and colour rebound at a 1-year control, even when measured by CIEDE2000 and Whitening indexes (WI and WI_D_), which according to the literature, reflect more precisely the rebound of whitening treatments^41^.

The presence of k21 with tetraethoxysilane as an anchoring unit evokes an organically modified, three-dimensional network due to the hydrolysis of the remaining silanol groups. This is followed by subsequent condensation to increase the Si–O–Si linkages^23,28^. The authors speculate that the antimicrobial used at the particular concentration may have affected the bleaching extract at 20 g concentration on changes in the colour. Condensation at a higher strawberry extract concentration have a greater shade variation than any 10 g samples alone. It is again speculated that light would be scattered and absorbed below the surface resulting in more variation in the colour measurements. Controlling colour is therefore more difficult when the number of layers is thickened or increased due to compact arrangement of particles with a 39minimal amount of entrapped air, in this case the silicate network, produced by k21. The baseline stain of coffee was clinically detectable and was used to randomize the specimens before the bleaching/staining cycles began.

For all the natural whitening products, the results obtained using the HPLC–UV method, the amounts of strawberry polyphenols were found in all 10 g samples tested were similar, except for the difference in intensities, with intensity higher for 20 g specimens. The precision obtained during HPLC/UV assays indicates an acceptable relative standard deviation (less or equal to 2.0%) with the regulated acceptable concentrations of 10 g as the ideal content formula. Based on the results, the behaviour of strawberries followed a trend. Furthermore, quercetin in extracts decreased in 10 g specimens (data not shown). Moreover, the variation of different polyphenol compounds was explained as improved extractability or increased concentration, leading to an improved detection.k21 organofunctional silane is an established compound with strong antimicrobial potency^23,26,29,30^. The classical method of disrupting the lipid bilayer of the bacterial cell by the quaternary ammonium silane is pivotal in disturbing the cytoplasmic contents. This is possible by the bonding of quaternary ammonium silane to the anionic sites on the bacterial membrane. This completely disrupts the osmoregulation of the bacterial cell leading to a massive leakage of protons and potassium ions. The positively charged quaternary nitrogen of the compound with hydrophobic tail interacts with the phospholipid acids in the bacterial membrane core as the changing the membrane from fluid to a liquid crystalline along with phospholipids (lipids A)^33^. This leads to the formation of a hexagonal arrangement^31^. As seen in the results, the number of microorganisms decreased significantly with k21 additions (Table 2) (p < 0.05), with 2%k21 showing the greatest decrease in bacterial colonization.

All bacteria were identified after evaluating bacterial colonization at the start of culture and after 7 days. The sol–gel method is a simple process of preparing the k21 molecule that pivots itself as an anchoring unit trialkoxysilane molecules forming the 3D network^32^ after the condensation is complete. The k21 molecule via Si–O linkages offers non-migrating microbiocidal function^33^, a surfactant activity, attracting bacterial organisms towards them (Fig. 1D). The selected strawberry extracts (10/20 g) have shown antibacterial activity against the lactobacillus bacterial stains. Moreover, according to the results of antimicrobial activity, there was no significant difference between the strawberry extracts (10/20 g) except the strawberry extracts modified with k21 precipitates. The extracts with k21 were the most effective in inhibiting the growth of lactobacillus. Therefore, the null hypothesis that there is no difference in antimicrobial potency between the commercial bleaching product and six different experimental strawberry extract gels was rejected. However, the antimicrobial activity of the extracts owes to formation of hydrogen bonds with water molecules within the bacterial wall due to the presence of phenolic acids, flavonoids, and tannins^34^. Also, there is coagulation of bacterial cell proteins leading to reduction of enzymes responsible for bacterial metabolism, essential for increase in cell division^35^. Therefore, such formulations could be considered as natural and safe. Thus, when compared to higher concentrations, the results of the current study showed that 2% k21 and polyphenol strawberry extracts with 10 g as the minimal concentration to achieve the desired efficacy could have overcome any shortcomings of existing bleaching agents.

Generally, the mature hydroxyapatite crystals form stable lattice domains that are lattice transformed. These have been identified by the 960 cm^−1^ Raman peaks which are characteristically assigned to ^3^PO_4_ antisymmetric stretching and the vibrational mode of the PO_4_ groups. Higher intensity peaks were observed in specimens treated with strawberry extracts, with 10 g exposure exhibiting the best spectrum confirming its higher mineral content. The change in intensity ratios is a confirmation of the change in surface properties. The 960 cm^−1^ peaks had a negative shift and significance with 20 g and Opalescence specimens. The lower the intensity of these inorganic components, the lower the value obtained in Raman readings. Table 3 shows the evolution of the peak area in the frequency range where the hydroxyapatite peaks are observed. While the absolute values are meaningless because they depend on experimental conditions, the evolution of these areas by varying just the number of defects provides interesting insights. There is a monotonic increase in the area with increasing concentration of the strawberry extract. This shows that the effect of increasing Raman change actually leads to more surface changes.

These enamel surfaces are prone to FIB beam damage, as one would expect from an organic or inorganic material. Because these enamel surfaces were not observed in the untreated samples using STEM, it is speculated that they are inorganic prisms onto which dissolved mineral precipitated after treatment. A systematic comparison of the microstructures of the treated samples and sound samples is currently underway. The interprismatic enamel crystallites can be seen to follow the curvature of the prism boundary and curve into the space between the prisms in strawberry extract specimens. However, it is impossible to tell from these STEM images whether these gaps are filled by the organic material that is thought to be present in the zone surrounding the prisms or if they are empty pores damaged by different bleaching agents. Despite this, the boundary region has a significant pore space when compared to the denser area inside the prism. At lower magnifications, the arrangement of prismatic and interprismatic enamel varies amongst different specimens. The prisms appear to be neatly arranged in 10 g strawberry extract specimens, in contrast to 20 g specimens where the prism boundaries are more open. This apparent confusion about the nature of the enamel surfaces between different specimens may have arisen as a result of the different uses of concentrations. The enamel crystallites (Fig. 4J–L), which appear black on the STEM micrographs, are unwavering than the crystallites of Opalescence in cross-section. The Opalescence treated crystallites appear to be broken (Fig. 4L) throughout the whole sample. It could also be possible that at these locations crystallites in Opalescence samples were broken out during sample preparation, but observing other specimen results treated with opalescence, it may be highly unlikely. In Fig. 4, it is clear that the crystallites fit together like puzzle pieces and take on irregular shapes after strawberry treatment. Furthermore, the crystallites appear to be in direct contact at times, though this may be due to crystallites overlapping in the third dimension, making interface analysis more difficult. The scanning electron microscopic images of the groups treated with strawberry extract alone suggested that it was safe for use on dental enamel, as no differences in enamel morphology were observed when compared to the group treated with Opalescence.

The structural characteristics of dental enamel observed in this study influence the material's mechanical behaviour. When a compressive load is applied to this structure in the x-direction, parallel to the prisms, it will have a high resistance because the crystallites are mostly aligned, and load can be transferred along them^39^. Load applied to strawberry treated structures demonstrated high resistance because the crystallites are mostly aligned and load can be transferred along them, resulting in a significantly higher (p < 0.05) apparent elastic modulus. Load transfer from one crystallite to the other is possible if the crystallites are connected by electrostatic interactions and H-bonds, involving energy dissipation via protein unfolding and bond breaking^36^. As a comparison, the XPS results for the specimens were consistent with what would be expected. In addition, the 10 g strawberry extracts exhibited weaker antioxidant activity than all the 20 g in this study, primarily due to the low content of phenolic compounds. Despite the lower antioxidant capability in comparison to the standards, the antioxidant potential of strawberry extracts in the preceding work is considerably higher when compared to the standards.

Many polyphenol antioxidants and therapeutic effects are thought to be linked to biologically active polyphenol components, such as flavonoids and phenolic acids, which have strong antioxidant properties^37^. Docking techniques are used in pharmaceutical research for a variety of purposes, the most common of which is virtual screening of large databases of available chemicals to select likely drug candidates. A study involving molecular docking and virtual screening was conducted to better understand the mechanism of binding and to identify the potency of the bleaching agents used (strawberry extracts). Using docking, hydrogen bonding between polyphenol hydroxyl groups has been studied. Out of the three chemical constituents, coumaric/malic acids and quercetin have the highest docking score (data not shown) and the most hydrogen bonds formed. According to the autodocking software results, coumaric/malic acids, quercetin, have a high affinity for the calcium of hydroxyapatite crystals. A combination of molecular docking, morphological characterization, and biochemical analysis, as well as the establishment of correlations between the binding obtained from these methods, would be a reasonable approach to characterizing the binding interactions between dietary polyphenols and the tooth structure.

The current study compared the ex vivo biocompatibility of all bleaching agents after 24 h of contact with hDPCs. To better simulate the clinical situation, the experimental groups were also applied to 0.4 mm thick enamel discs along with exposure in conditioned media. According to the results of the cytotoxicity assay, a higher percentage of hDPCs remained viable after being exposed to group 1 and 2 at all-time intervals. Groups 3 onwards showed significant reduction (> 50%) when cells were exposed through conditioned media. However, with increasing strawberry concentrations and exposure times through enamel surfaces, there was less cell morphology change observed with small-sized cells with open blebs seen amongst cells exposed to Opalescence (Fig. 2H). These were significant areas, may have corresponded to the toxic effects of the Opalescence gel. The change in cell morphology observed in this study could be attributed to the opalescence diffusion inside the cell. Therefore, it seems liable to assume that the use of strawberry extracts should recommended as bleaching projects because of its lesser cytotoxic effects rejecting the null hypothesis. This renders 10 g strawberry extracts a safer bleaching product. Further studies are required to elucidate the mechanisms responsible for the more favorable biocompatibility of strawberry extracts. Finally, this study found that 10 g strawberry extracts, whether combined or not with HA, was effective at bleaching artificially stained specimens. These results allowed us to infer that 10 g strawberry extracts may be combined with low concentrations of 2%k21 for the attainment of satisfactory and immediate bleaching outcomes with antimicrobial potency while avoiding maximum surface changes. However, increase in K21 concentrations maybe accompanied by increases in cytotoxicity.

## Conclusion

The efficacy demonstrated in this study indicated that all bleaching solutions resulted in perceptible colour difference. All the groups showed whiteness values with no statistically significant difference among the test groups and control group indicating the combination of HA and K21 along with strawberry supernatant can result in the development of an effective bleaching agent. The 10/20 g specimens exhibited thinner biofilms with no distinct aggregates, as the concentration of strawberry was increased, indicating a dominant role for bacterial biofilm removal and non-adherence while performing the bleaching procedure in presence of K21. The underlying results related to antimicrobial action by k21 against bacterial growth show strong potential for the development of safer bleaching products.

## Materials and methods

**Figure 9:**
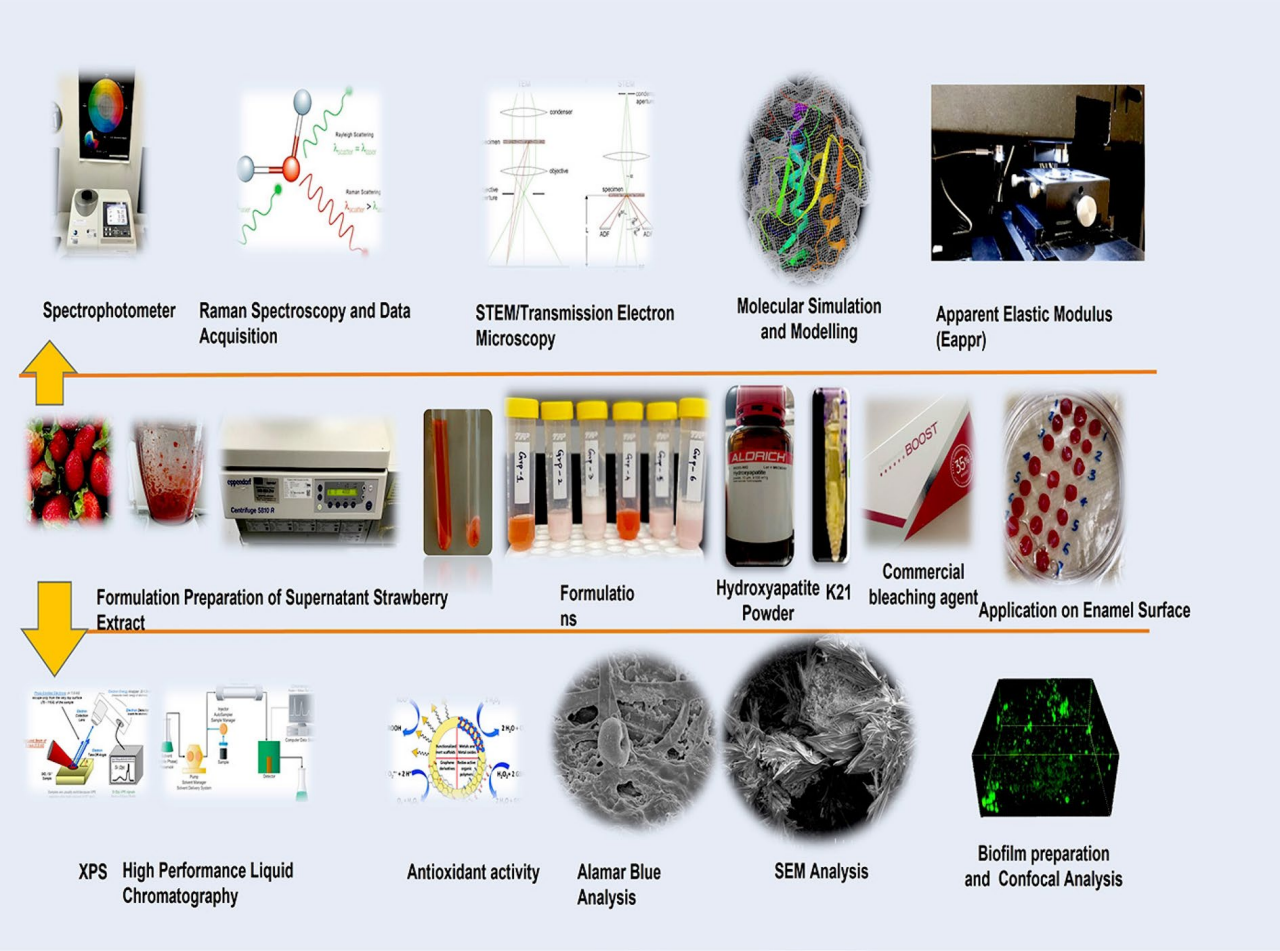
Schematic representation of the experiments performed on enamel coated with different bleaching agents.

A priori, all experiments were approved by the Ethical Committee at International Medical University, following the use of Grant #489/2020. The licensing committee approving the experiments, included all relevant details; confirming that all experiments were performed in accordance with relevant guidelines and regulations. The use of plants in the present study complies with international, national and/or institutional guidelines. Sound human incisors (*n* = *70*) were collected (25–40 years) after ethical approval (489/2020-IA562). The present study’s experimental design is depicted in Fig. 9. The samples were randomized to ensure that results obtained from the groups approximated for the entire group of sampling. The sample size for analysis of specimens was derived by the following equation keeping the power of study equal to 90% and level of significance equal to 5%. The enamel specimens (3 × 3 × 2 mm^3^) for all experiments were prepared using ISOMET Low-Speed Saw (Isomet, Buehler, Lake Bluff, IL, EUA) with two diamond discs (Extec Corporation, XL-12205, Enfield, CT, EUA). After preparation, the specimens were cleaned by placing them for 15 min in an ultrasonic bath (T7 Thornton, Unique Ind. e Com. Ltda., São Paulo, SP, BR). The samples were randomized to ensure that results obtained from the groups approximated for the entire group of sampling. The sample size for analysis of specimens was derived by the following equation keeping the power keeping the power of study equal to 90% and level of significance equal to 5%.

### k21 (Quaternary Ammonium Silane) powder

The Quaternary Ammonium Silane (CaC2 enriched; ethanol-based solution form) was supplied by KHG fiteBac Technology, Marietta, GA, USA.

### Formulation preparation of supernatant strawberry extract

Fresh strawberries (600 g), without skin removal (*garden strawberry; Fragaria* × *ananassa; hybridization between two octoploid strawberries*), bought from Mini Cameron Highlands Garden Genting (Genting Highland, Pahang), were crushed, and homogenized with a blender (Philips, HR 2115, Indonesia). Ten grams of strawberry preparations were dissolved in 50 ml of distilled water for 2 h at room temperature (23 °C). The solutions were centrifuged at 4000 rpm (Fig. 9) for 10 min (4 °C), leaving the precipitate behind (supernatant strawberry extract). The following compilations were finalized to formulate six groups apart from the in-office bleaching product—Opalescence Boost 35% (505 West Ultradent Drive, Jordan) used for experimental protocols: Group 1: supernatant strawberry (10 g) extract only < Group 2: supernatant strawberry (10 g) extract + 15% HA < Group 3: supernatant strawberry (10 g) extract + 15% (HA-2%K21) < Group 4: supernatant strawberry (20 g) extract only (made from 20 g of mashed strawberries) < Group 5: supernatant strawberry (20 g) extract + 15% HA < Group 6: supernatant strawberry (20 g) extract + 15% (HA-2%K21) < Group 7: In-office bleaching product—Opalescence Boost 35%.

### Spectrophotometric methods

#### Preparation of samples

Seventy sound anterior teeth were selected devoid of caries, crack, fractures, or staining. The teeth were cleaned from calculus and debris using an ultrasonic scaler (Dentsply Sirona). Under water cooling, the specimens were decoronated at the cementoenamel junction with a rotating diamond disc (SIDIA-FLEX). Using a digital caliper, the crowns were sectioned mesiodistally, with each center of the blocks measuring between 0.8 and 1.1 mm. Each specimen was prepared individually by submerging the sectioned tooth in clear chemically cured acrylic resin (Temp, Istanbul, Turkey) with the labial surface exposed in a round silicon mold of 1.7 cm diameter to ensure that the same area will be measured each time. After preparation, the samples were cleaned using an ultrasonic scaler. After that, the labial surfaces were flattened using with silicon carbide (SiC, FEPA P#1000, Struers) paper of #600 and #1200 grit under constant irrigation.

#### Staining of the specimens

All the samples were submerged in black coffee (Nescafé Classic Tin 1 kg: the black coffee was prepared by (one user) dissolving 4 g of coffee in 200 ml of hot water). The specimens were immersed for 1 week with samples washed after every 24 h and the staining solution replenished^16,38^. After staining, all specimens were subjected to cleaning with pumice and the colour measurements were performed. The colour measurement of the teeth samples was performed using CIE L* a* b* system with a spectrophotometer (Konica Minolta CM-5, Osaka, Japan). All the colour measurements were made thrice on the middle third area of each block toothsurface, and the mean value was calculated.

### Application on enamel surface and colour measurement

Prepared strawberry extracts were applied using a thin brush (synthetic acrylic, No. 2) on prepared blocks with a contact time of 8 h/day for 5 consecutive days^16^. After application, all specimens were thoroughly rinsed with water and stored in reverse osmosis water (RO) until the next bleaching cycle. The prepared strawberry extracts were stored in refrigerator (Samsung (RT20FARVDSA/ME) 220L Digital) after application. Application of Opalescence Boost 35% was performed according to manufacturer's instructions. Samples of all the groups were immersed in RO water.

Colour measurements were performed immediately after bleaching. Colour difference (∆E00) was calculated using an Excel spreadsheet implementation of the CIEDE2000 colour difference formula provided by Dr. Klaus for Program of Colour Science/ Munsell Colour Science Laboratory from Rochester Institute of Technology^39^. The colour change was measured between after staining and after bleaching (∆E00), CIEDE2000 colour difference (ΔE00) and the clinical perceptibility and acceptability thresholds were set at 0.8 and 1.8, respectively according to the previous study^40^.

The color change was calculated as a function of the whiteness index (WI_D_). The WI_D_ of the specimens after the staining (WI_D_1) and after the bleaching (WI_D_2) was calculated using the following formula^41^:

$${\text{WI}}_{{\text{D}}} = 0.{\text{511L}}* - {2}.{\text{324a}}* - {1}.{1}00{\text{b}}*$$

where WI_D_ is the whitening index, 0.511 is the constant of lightness, 2.32 is the constant for the coordinates from green to red, and 1.100 is the constant for the coordinates from blue to yellow. ∆WI_D_ which is the difference between the whiteness after staining (WI_D_1) and whiteness after bleaching (WI_D_2) was calculated using the following formula:

$$\Delta {\text{WI}}_{{\text{D}}} = {\text{WI}}_{{\text{D}}} {2} - {\text{W}}_{{{\text{ID}}}} {1}$$

### Lactobacillus biofilm and confocal analysis

To inhibit bacterial growth, sound human third molars (n =) were stored in 0.2% sodium azide at 4 °C, and the teeth were used within 1 month of extraction. The experimental study protocol was pre-approved by Institutional Review Board (Grant number #489/2020). The enamel specimens (3 × 3 × 2 mm^3^) were cut with an ISOMET Low-Speed Saw (Buehler, Lake Bluff, IL, EUA) and two diamond discs (Extec Corporation, XL-12205, Enfield, CT, EUA). Following preparation, the specimens were cleaned in an ultrasonic bath (T7 Thornton, Unique Ind. e Com. Ltda., So Paulo, SP, BR) for 15 min. The bleaching solutions were vortexed repeatedly until the solute was completely dissolved. Each time the substrates were scheduled for treatment, new treatment solutions were prepared. After application, the enamel specimen blocks were placed in a microplate and co-cultured with an equal volume of bacterial solutions. For single-colony lactobacillus (ATCC 15578™) experiments, a 300 µL aliquot of each bacterial culture was mixed and transferred to the enamel discs individually. Specimens were placed in sterile 24-well tissue culture plates (Thermo Fisher Scientific Inc.) and incubated for 3 days at 37 °C in an anaerobic chamber, to allow bacterial adhesion. The biofilms were first incubated for 120 min at 37 °C in an orbital shaker incubator at 75 rpm before moving on to the adhesion phase. Under anaerobic conditions, the biofilms (107 CFU/mL) were allowed to grow for 3 days (85% nitrogen, 10% hydrogen, and 5% carbon dioxide). Every day, the medium was replaced with a new solution, and the pH of the culture media (7.400.2) was measured every 24 h.

The microplate containing substrates and bacteria were incubated in an anaerobic chamber (85% N2, 10% H2, 5% CO_2_) for 24 h. After 24 h, the spent BHI media was withdrawn to replenish the substrates with biofilms from fresh BHI media. The 24-h treatment was followed by 7 days of bacterial growth. The biofilm-covered substrates were then removed and washed in PBS before being characterised.

Lactobacillus biofilm was examined using a 0.1 wt% aqueous solution of bleaching agents. The specimens were examined with a confocal laser scanning microscope (CLSM, Leica Fluoview FV 1000, Olympus, Tokyo, Japan), which was outfitted with a 60/1.4 NA oil immersion lens and was illuminated with a 488 nm argon/helium laser beam and a 633 nm krypton ion laser. The modes of reflection and fluorescence are both supported. Using a photomultiplier tube, reflected and fluorescence signals were detected to a depth of 20 m and then converted to single-projection images for better visualisation and qualitative analysis. bioimageL software (v.2.0, Malmö, Sweden) was used to examine the fluorescent images of the biofilm at different stacks. These stacks represent the structure of the biofilm with green and red stained colonies on a two-dimensional x–y section based on MATLAB-written colour segmentation algorithms. The live and dead bacteria percentages were later calculated.

### SEM analysis

A JCM-5700 Scanning Electron Microscope was used to image all samples (JEOL USA, Peabody, MA, USA). Under a high vacuum at 6 kV, gold-coated specimens were imaged with an 8 mm working distance and a 30 m objective lens aperture. Images were collected using a secondary electron detector, with an acquisition time of 160 s per image and a resolution of 2560 × 1920 pixels adjusting the acceleration voltage to 4 kV. Multiple magnifications were used to capture SEM images. The diameters of dental pulpal cells were measured using the Image J straight line tool package. The length measurements were calibrated using the image's scale bars and Image J analyse/set scale function.

### Raman spectroscopy and data acquisition

Raman spectra were collected using a Renishaw RM-2 Raman microscope system with an Olympus (BH-2) 20 objective lens (ULWD). The specimens were excited with 785 nm radiation from a diode laser at a power of 5 mW. The spectra were collected over a 200 cm^−1^–3200 cm^−1^ range, with 40 scans coadded to produce the final spectrum. For baseline correction, a fifth-degree polynomial function was used. Peaks for v1 v3 phosphate vibration were identified on enamel discs (n = 10), with three sub-bands (1060, 1030, and 960 cm^−1^). 2–3 points were selected to draw a baseline using the “baseline” tool of the software, to reduce the background from the fluorescent groups. Subsequently, using the tool of “baseline” again, 10 points were taken to draw a smooth curve along the bottom of the scattering peaks. The peak areas with changes were highlighted in the Table 3.

Steps used to scan at different polarization angles include ∆β = 15°, from β = 90° to β =  − 90°. For calculation, the following equation was fitted in Matlab 7.5 (MathWorks Inc., Natick, MA, USA).

1 $$I = a\left( {1 + b\left( {cos\left( {2\left( {\beta - c} \right)} \right)} \right)} \right)$$

*I* is intensity response of hydroxyapatite; hydroxyapatite average intensity of all scans done on enamel surface; *b* fitting curve amplitude; *β* laser polarization angle; *c* phase shift^42^.

### STEM/transmission electron microscopy

Enamel slices were cut from the labial or palatal sections of the teeth for FIB (Focused Ion Beam) specimens using a Buehler Isomet 4000 precision saw (Buehler, Esslingen, Germany) while the teeth were irrigated with water. Following that, smaller pieces were cut as described in “Results”. Conductive silver was used to bond the samples to electron microscope stubs. After grinding the specimen with SiC papers ranging from 1200 to 4000, the surfaces were vacuum dried, and sputter coated with a thin gold layer for 60 nm. The lamellae were created using a dual-beam FIB system (FEI Helios G3 UC, FEI Deutschland GmbH, Frankfurt/Main, Germany) by cutting the enamel prisms perpendicular to the lamella. The lamella electron was then transparent after several thinning steps with currents of 0.79 nA, 0.23 nA, 80 pA, and finally 40 pA. STEM images were captured using collected inelastic and elastic scattered electrons and a high angle annular dark-field (HAADF) detector (FEI Deutschland GmbH, Frankfurt/Main, Germany).

### High performance liquid chromatography

Agilent 1200 HPLC system (Santa Clara, CA, USA) was used for analysis. The instrument was equipped with a diode array detector. The injection volume within the reverse phase Nucleosil C18 Macherey–Nagel column was 10 μL, with detection performed at 361 nm. A gradient elution water dispersion (solvent) was used as a mobile phase with hydrogen peroxide at 25 °C with a flow rate set at 1.0 mL min^−1^. The chromatograph (CTO-10AS) and UV detector (SPD-20A) were performed. Peak areas of analytical signals were used to determine and quantify polyphenols in the HPLC.

### Apparent Elastic Modulus (Eappr)

Using a G200 Nano-indenter, nano-indentation testing was performed at the desired time intervals (baseline) (Agilent 7 Technologies, Santa Clara, CA, USA, MIMOS Research lab). A Berkovich diamond-indenter with a tip radius of 40 nm was used at a constant strain rate of 0.05 s^−1^ and a hold-time of 5 s to achieve a maximum indentation depth of 70 nm at a load range of 400–500 N. Ten indentations were made on designated spots with a lateral spacing of 400 nm for each specimen (n = 7/group). The elastic modulus (Er) was calculated.

### XPS (X-ray photoelectron spectroscopy)

AXIS Ultra DLD electron spectrometer was used to collect XPS spectra (Kratos, UK). Specimens were exposed to 180 W X-rays and monochrome Al K (1486.6 eV). A survey spectrum of 0–1200 eV was collected at a high-resolution spectra of C 1s, P 2p, and Ca 2p regions with pass energies of 160 eV and 40 eV, respectively, and data was analysed using CasaXPS Software (Casa Software Ltd, United Kingdom).

### Antioxidant activity

The antioxidant activity was determined using 300 μL aliquots of strawberry extracts (all groups or specific groups) and added to 1 mL of DPPH at a concentration of 25–150 μg mL^−1^. The mixture in Eppendorf tubes were incubated in the dark at room temperature for 40 min. Solutions of 200 μL and 1 mL of DPPH were used as controls. The absorbance was recorded at 518 nm and DPPH inhibition calculated using the equation:

2 $${\text{Radical inhibition}}\left( \% \right) = \left[ {\left( {{\text{A control}}{-}{\text{A}}_{{{\text{sample}}/{\text{reference}}}} } \right) \times {1}00} \right]/{\text{A}}_{{{\text{control}}}}$$

### Molecular simulation and modelling

The present study was undertaken to determine the molecular interactions between polyphenols and enamel by computational molecular docking studies. Molecular docking studies were carried out on crystal structures. The data on hydroxyapatite was obtained from http://rruff.geo.arizona.edu/AMS/result.php and drawing done using Marvin, ChemAxon. The crystals were visualised using VESTA 3 for volumetric and morphology data^43^. Glide (Standard precision (SP) mode and Extra precision (XP) mode) and induced-Fit docking modules of Schrodinger 2020-2 were used to determine the interactions between bioactive compounds and enamel hydroxyapatite. From the binding energy and interaction studies, all the compounds were determined for interactions. The compounds were prepared for molecular docking studies using protein preparation wizard with OPLS-3e force field at pH 7.20 ± 0.20 and the other default settings. For further confirmation, the binding efficacy of the compounds were done with induced-fit docking studies using ‘Induced-Fit docking’ module within the range of 5 Å of the receptor made flexible. In general, induced-fit docking provides better insights on binding interactions and efficacy. The poses with highest negative docking scores are shown in the results^23^.

### Alamar blue analysis

#### Dental pulp stem cells

Human dental pulps stem cells (hDPSCs) were commercially obtained from Lonza (Lonza Walkersville, Inc. Walkersville, MD 21793-0127 USA). The cells were cultured using the manufacturer-recommended Bullet media kit (PT-3005) containing DPSC basal media and growth supplements including L-Glutamine, Ascorbic acid, Gentamicin/Amphotericin-B, along with 10% DPSC specific fetal bovine serum. The hDPSCs were sub-cultured, expanded, and trypsinized as required. Cells cultures in 3rd–4th passages were used in the experiment.

### Preparation of the conditioned media

The conditioned media was prepared following the method described by Cavalcanti et al.^44^. Briefly, 0.2 g/mL (200 µL) of test reagents were added to the DPSC culture media and incubated at room temperature for 30 min. Further, the media was filtered using 0.22 µ cellulose acetate syringe filters (Steriletech, Kent, Washington, USA) to collect and store the conditioned media for each test reagent.

### Cytotoxicity assay

The cultured hDPSCs were seeded at 2 × 10^4^ cells/well in a 96-well flat bottom plate, and cells were allowed to settle for 24 h. Then, the cells from all the groups were treated with the prepared conditioned media for 1, 6, 12 and 24 h. Cytotoxicity was determined using Alamar blue, a resazurin dye(manufacturer) that functions as a cell health indicator using the reducing power of living cells to measure cytotoxicity quantitatively. Briefly, After the hDPSCs were treated for a certain period (1, 6, 12, and 24 h), 10 µL of resazurin dye was added to each well and incubated for 8 h. Untreated cells exposed to regular culture media were used as control. Later, absorbance from the cells was read at 570 nm and 600 nm using a spectrophotometer.

### Seeding hDPSCs on prepared Enamel Discs

The hDPSCs were expanded further, and cells from 4 to 5th passages were seeded on one side of the prepared enamel discs after bleaching applications. The enamel discs were carefully placed inside appropriate wells of a 96 well plate containing 100 µL of prepared conditioned media. The cells were allowed to grow and reach approximately 80% confluence. Further, the cells were fixed using 2.5% glutaraldehyde. All samples were imaged using a JCM-5700 Scanning Electron Microscope (JEOL USA, Peabody, MA, USA).

### Statistical analysis

All of the statistical analyses were performed by using IBM SPSS Statistics for Windows, version 21(IBM Corp., Armonk, NY, USA). Unless stated otherwise, the *p* value for all statistical tests were stated as *p* < *0.05* as all datasets were subjected for homoscedasticity and normality. For colour analysis, descriptive statistics were performed for Δ*E*00 and ΔWI_D_ values and One-way ANOVA test was used to detect statistical differences between the groups, followed by pairwise comparisons post hoc Bonferroni. Where datasets were normally distributed, a one-way ANOVA was performed followed by Tukey post-hoc comparison for mean percentage values of bacteria, mechanical properties, and other Raman crystal range and XPS values. The post-hoc test was performed after statistically significant result was obtained. Therefore post hoc tests explored between multiple k21 and control groups controlling the experiment-wise error rate.

### Ethics approval and consent to participate

The project was approved by Institutional Review Board of International Medical University Kuala Lumpur. Informed consent, written, was obtained from all participants donating extracted teeth for the lab in-vitro experiments approved by the ethics committee.

## Data Availability

The datasets generated and/or analysed during the current study are not publicly available due to confidentiality but are available from the corresponding author on reasonable request.
